# Transcriptional regulation in cardiovascular aging

**DOI:** 10.3389/fcvm.2026.1752877

**Published:** 2026-04-15

**Authors:** Yiguang Fan, Chao Song, Fangnan Feng, Qing Xun, Yuhao Lu, Chunquan Li

**Affiliations:** 1 The First Affiliated Hospital & Hunan Provincial Key Laboratory of Multi-Omics and Artificial Intelligence of Cardiovascular Diseases, Hengyang Medical School, University of South China, Hengyang, Hunan, China; 2 The First Affiliated Hospital, Institute of Cardiovascular Disease, Hengyang Medical School, University of South China, Hengyang, Hunan, China; 3 Department of Biochemistry and Molecular Biology, School of Basic Medical Sciences, Hengyang Medical School, University of South China, Hengyang, Hunan, China

**Keywords:** aging, cardiovascular diseases, epigenetic, transcription factor, transcriptional regulation

## Abstract

Cardiovascular diseases (CVD) stand as the primary cause of mortality and form the most prevalent disease category. Numerous studies have demonstrated that aging serves as a crucial pathogenic factor in the progression of CVD. Nevertheless, the molecular mechanisms underlying cardiovascular aging have not been fully elucidated. Research in various aging models has consistently shown that aging is attributed to the dysfunction of a complex transcriptional regulatory network that maintains the body's health, tissue homeostasis, and stress resistance. Transcription factors and chromatin regulatory factors are involved in almost all cellular activities, and an increasing amount of evidence indicates that as key regulatory elements, transcription factors and chromatin regulators control cellular senescence by regulating the transcription of related genes. Therefore, the aim of this review is to summarize the specific role of transcriptional regulation in the mechanism of cardiovascular aging, providing new strategies for the treatment of CVD

## 1 Introduction

CVD, a group of disorders affecting the heart or blood vessels, are the primary cause of death worldwide, with an immense impact on patient quality of life and disability. Among these diseases, age is a crucial risk factor that causes the gradual degradation of the structure and function of the cardiovascular system (1). With the increasing severity of the aging population trend, aging-related diseases, particularly those of the cardiovascular system, are becoming more prevalent. Clinical studies have shown that the mortality rate of CVD increases exponentially with age. The aging-related alterations in intracellular homeostasis make the hearts and blood vessels of the elderly more vulnerable to pathophysiological conditions. Exploring the pathophysiological mechanisms related to aging is helpful for reducing the mortality rate of age-related diseases of the cardiovascular system (2).

Aging is a biological process marked by the progressive deterioration of cellular and tissue functions over time, which contributes to the rising incidence of age-related diseases. It represents a significant risk factor for numerous pathological conditions, including hypertension, atherosclerosis, and heart failure (3). The aging process is mainly related to the accumulation of senescent cells (4). Cellular senescence is a biological process characterized by the progressive loss of cellular structure and function, ultimately resulting in cell cycle arrest rather than immediate cell death. It can be induced by a variety of stimuli, including activation of intracellular signaling pathways, oxidative stress from free radicals, dysregulation of gene expression, and alterations in the extracellular matrix microenvironment. These stressors disrupt normal cellular homeostasis and functional integrity, leading to irreversible growth arrest. Cellular senescence is closely associated with multiple domains in biology and medicine, particularly aging research, age-related diseases, and regenerative medicine (2, 5, 6).

Transcriptional regulation is crucial for all aspects of cellular function and determines the fate of cells. They are selectively expressed in particular cell types and regulate the gene expression pattern. They recognize specific DNA sequences called response elements or transcription factors (TFs) binding sites and activate or inhibit gene expression (7). TFs Sox9 plays a crucial role in vascular development. Sox9 has a decisive influence on the differentiation direction of vascular smooth muscle cells (VSMCs), determining their differentiation by regulating the expression of the smooth muscle marker gene *α*-SMA (alpha smooth muscle actin) (8). In addition to TFs, the chromatin state composed of various histone marks and DNA methylation also participates in the regulatory process of the spatio-temporal expression of genes (9). For example, the chromatin remodeling complex SWI/SNF (Switching defective/sucrose non-fermenting Factors) regulates cell lineages by maintaining enhancer activity (10). Given its crucial role in cell functions, we believe that the phenomenon of age-related changes in TFs and chromatin state is not only a core feature of the aging process but also an important hub connecting multiple hallmarks of aging (11). Aging is accompanied by a significant decline in transcriptional regulatory capacity, and this change is particularly prominent in the cardiovascular system, becoming an important factor driving cardiovascular aging and related diseases. For example, during the development of atherosclerosis, abnormal epigenetic modifications of key genes such as ERG, KLF4, and APOE are closely associated with disease stages (12, 13). We consider transcriptional regulation to be an important component of the mechanism of cardiovascular aging. Therefore, in-depth research on the role of transcriptional regulation in the mechanism of cardiovascular aging can help reveal its pathophysiological basis and provide new directions for the prevention and treatment of aging-related CVD. This article systematically reviews the molecular mechanisms by which dysregulation of transcriptional regulation leads to cardiovascular aging. We focus on aging-related CVD and delve into the central role of TFs and Epigenetic factors in the mechanism of cardiovascular aging.

## 2 The molecular mechanism of transcriptional regulation

**Figure 1:**
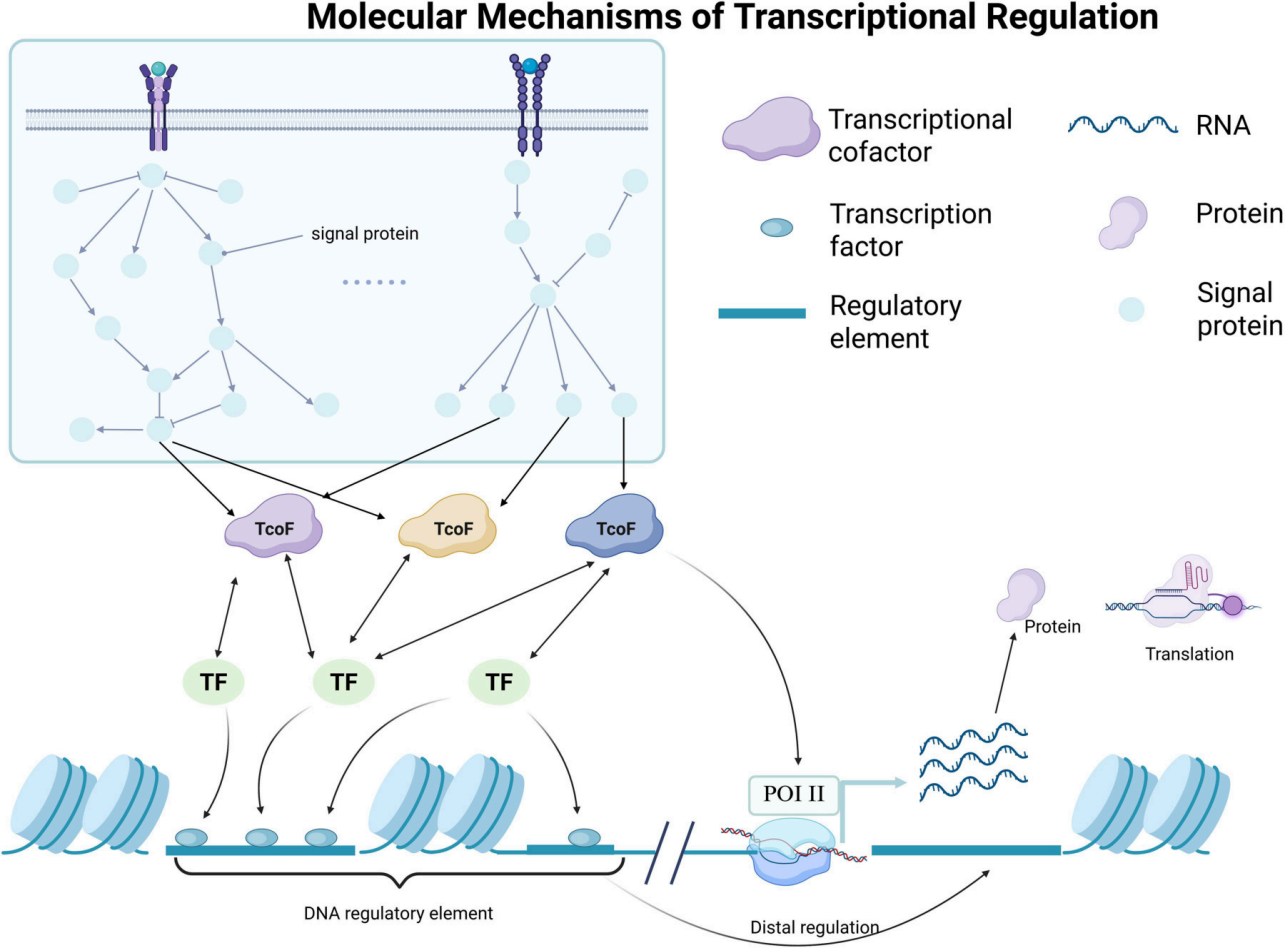
The mechanism of transcriptional regulation. TFs and Tcofs are recruited to the enhancer region, thereby transferring signals to the promoter and playing crucial roles in transcriptional regulation and gene expressio.

As the core mechanism of gene expression, transcriptional regulation involves the dynamic interaction of TFs, cis-acting elements and chromatin structure. This section will systematically elaborate the molecular basis of transcriptional regulation from three dimensions: the structural and functional characteristics of TFs, the regulatory mechanism of their activity, and their integration with epigenetics (Figure 1).

### 2.1 Structural and functional characteristics of TFs

TFs regulate the activity of RNA polymerase II in a gene-specific manner, thereby regulating the expression of related genes. The vast majority of TF contain two domains that are essential for regulatory function: a sequence-specific DNA-binding domain and a transcriptional activation/repression domain (14, 15). DNA-binding domains confer upon TFs the capacity to recognize specific DNA sequences. Common structural motifs encompass zinc finger structures, leucine zippers, helix-loop-helices, and other elements. These domains bind to the major or minor groove of the DNA double helix via hydrogen bonding and hydrophobic interactions, facilitating the specific recognition of promoter or enhancer regions. The transcriptional activation/repression domain is accountable for interacting with a diverse range of cofactors to form large multi-subunit protein complexes (16). These cofactor complexes can directly activate or inhibit the activity of RNA polymerase II on the one hand, and promote or inhibit the transcription of related genes by changing the chromatin structure on the other hand. In addition, certain TFs also possess unique “pioneer TFs” functions. These factors bind to densely packed chromatin regions and modify the chromatin structure by mediating nucleosome remodeling and histone modifications (17).

### 2.2 Regulation mechanism of TF activity

The activity of TF is subject to multi-level and multi-dimensional regulation to ensure the accuracy of gene expression in both space and time. At the level of DNA binding specificity regulation, TF bind to DNA promoter sequences or distant enhancer elements in a sequence-specific manner. These regulatory elements can span a large genomic region and achieve precise regulation of the target gene promoter. The binding affinity of TFs to DNA is dynamically influenced by various factors, including the conservation of the DNA sequence, chromatin accessibility, and the post-translational modification status of the TFs themselves (18). At the regulatory level of upstream signaling pathways, the expression and activity of TFs are regulated by a variety of upstream signaling pathways. Extracellular stimuli activate intracellular signaling cascades through membrane receptors, ultimately acting on TFs to achieve precise transmission from extracellular signals to gene expression in the nucleus (19). At the regulatory level of post-translational modifications, the activity of TFs is affected by phosphorylation, acetylation, ubiquitination, and SUMOylation, which can affect DNA binding ability, nuclear localization, protein stability, and interaction with other cofactors. For example, phosphorylation of STAT3 would enhance its transcriptional activity, whereas ubiquitination would target it for proteasomal degradation (20). Notably, these post-translational modifications are often a direct result of the action of upstream signaling pathways. This multi-level regulatory mechanism ensures that cells can accurately respond to changes in the internal and external environment and maintain homeostasis.

### 2.3 Chromatin structure and epigenetic modifications in transcriptional regulation

In eukaryotic cells, chromosomes are tightly packed into chromatin structures, with nucleosomes acting as the basic structural units. Each nucleosome consists of approximately 146 bp DNA wrapped around an octamer of histones consisting of core histones H3, H4, H2A, and H2B, linking histone H1 to bind to entry and exit sites of the DNA to stabilize the structure. The N-terminal tail of histones can undergo a variety of post-translational modifications, including methylation, acetylation, phosphorylation, ADP-ribosylation, and ubiquitination, and these modifications are catalyzed by specific epigenetic modifiers (21). These modifications are reversible, and the existence of different modification isoforms leads to different spatial arrangement of chromatin, which affects the interaction of DNA with RNA polymerase II and TFs. Histone modification, as a heritable epigenetic mark, can cause changes in gene expression without changing DNA sequence, and finely regulate gene transcriptional activity (22). In addition, TFs can actively alter local chromatin structure by recruiting histone modifying enzymes and nucleosome remodeling complexes. For example, the activation domains carried by TFs can recruit histone acetyltransferases (HATs), promote histone acetylation, relax chromatin structure and increase DNA accessibility. The repressor domain recruits histone deacetylases (HDACs), condenses chromatin and represses gene transcription. The sequence specificity of TFs, their ability to respond to upstream signaling pathways, and their function in recruiting epigenetic modifying enzymes collectively determine their central role in the regulation of gene expression (17).

## 3 Molecular mechanisms of cardiovascular aging

**Figure 2:**
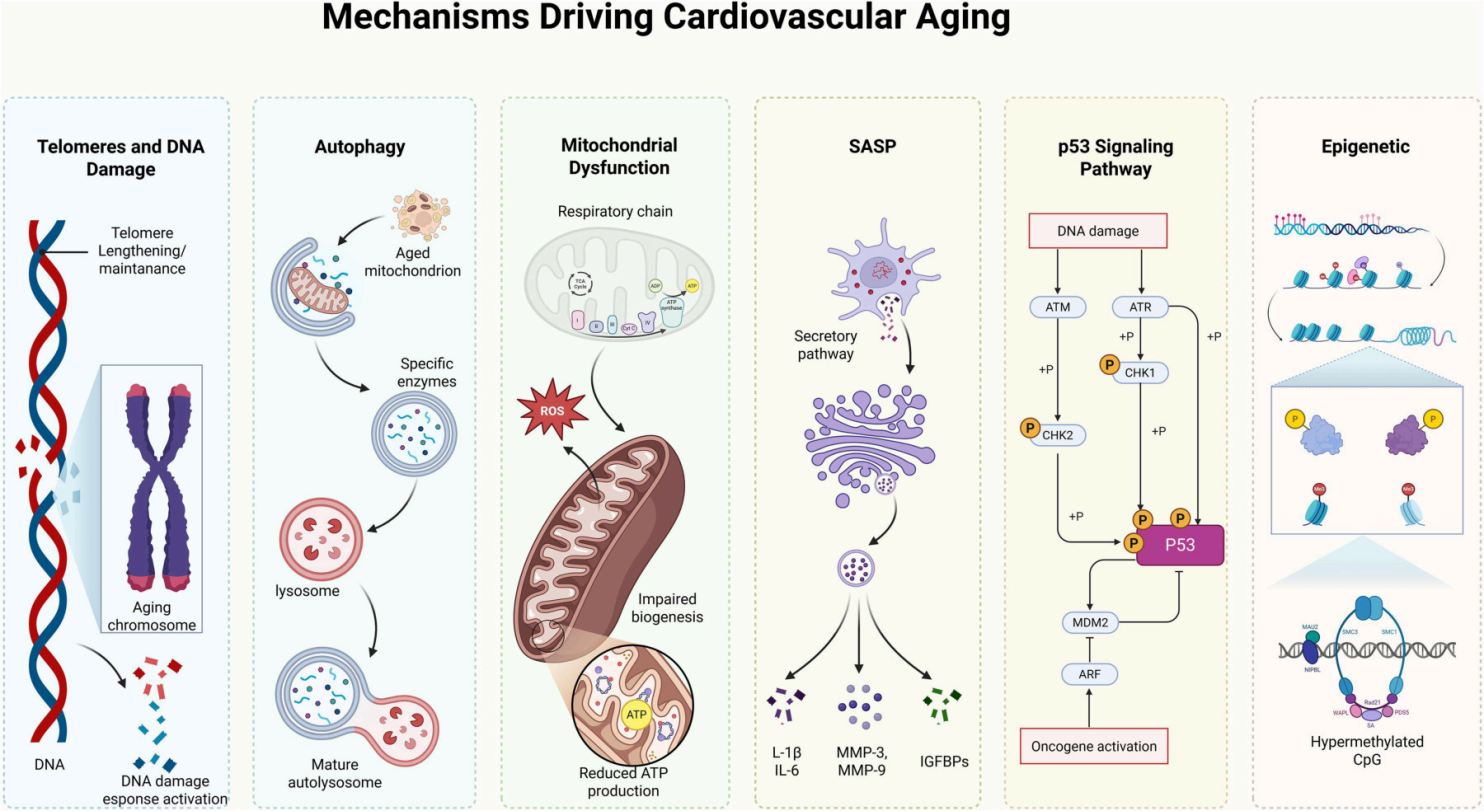
Molecular mechanisms of cardiovascular aging. Mechanisms that drive cardiovascular aging include the telomeres and DNA damage, autophagy, mitochondrial dysfunction, SASP, the p53 signaling pathway, and epigenetic changes.

In this section, we broadly discuss molecular mechanisms of senescence that can occur across diverse cells or tissues (Figure 2).

### 3.1 Telomeres and DNA damage

Aging causes the gradual wear and tear of telomeres. Telomeres are tandem repeat sequences at the ends of chromosomes, and their function is to protect chromosomes from degradation and end-to-end fusion. The shelterin complex can recognize the telomere structure, thereby preventing the DNA damage response mechanism from recognizing and processing telomeric DNA. During each cell cycle, telomeres gradually shorten. When the telomere length reaches a critical value, shelterin proteins can no longer be recruited to protect the DNA loop structure, thus activating the DDR system and triggering cell cycle arrest (23). Telomere shortening is a natural process of aging and can lead to replicative senescence, which is particularly evident in non-myocardial cells. Telomere maintenance and the senescence of endothelial cells and VSMCs associated with telomere attrition are regarded as part of the pathogenesis of degenerative vascular diseases (24).

### 3.2 The p53 signaling pathway

The induction of cellular senescence can be achieved by activating the p53/p21/p16/Rb signaling pathway, ultimately leading to cell cycle arrest. p53 is a TF that regulates genes related to metabolism, autophagy, DNA damage response, cell cycle, and apoptosis (25). Its activity is regulated by various post-translational modifications, including ubiquitination, phosphorylation, and acetylation. p53 positively regulates p21 [a cyclin-dependent kinase (CDK) inhibitor], and p21 is a key factor in p53-mediated cell cycle arrest at the G1/S or G2/M phase. p21 inhibits cell apoptosis by binding to caspases, thereby promoting the maintenance of the cellular senescence state (26). The p16/Rb signaling pathway is regulated by the inhibitory activity of p16. p16 binds to CDK4/CDK6 to prevent the phosphorylation of the Rb protein, thereby triggering cell cycle arrest at the G1/S phase (27).

### 3.3 Mitochondrial dysfunction

Mitochondrial dysfunction leads to CVD through apoptosis and senescence (28). Mitochondrial dysfunction and increased ROS production can promote DNA damage, including chromosomal and mitochondrial DNA. Senescent cells often exhibit overly elongated mitochondria due to the imbalance of mitochondrial fission and fusion proteins. The specific mechanisms are as follows: a relatively lower level of mitochondrial fission proteins (such as Fission, mitochondrial 1, FIS1 and dynamin-related protein 1, DRP1), or an increased expression of fusion proteins (such as mitofusin 1/2, MFN1/2, and optic atrophy 1, OPA1) can promote mitochondrial over-elongation and related senescence phenotypes (29, 30). The decline in mitochondrial function may lead to the production of reactive oxygen species (ROS), thereby affecting the inflammatory state and metabolism of cells. In addition, dysfunctional mitochondria may release their contents (such as mtDNA) into the cytoplasm. ROS and mitochondrial contents may synergistically activate the innate immune signaling pathway, leading to a further increase in IL-6, thus exacerbating mitochondrial dysfunction (31).

### 3.4 Composition and function of the senescence-associated secretory phenotype (SASP)

SASP consists of a series of cytokines, including pro-inflammatory cytokines, growth factors, chemokines, and matrix remodeling enzymes (32). SASP secretes various pro-inflammatory factors that affect cell and tissue biology. The soluble signaling factor interleukin-6 (IL-6): Its expression is considered to be directly regulated by the DNA damage response (DDR) and is independent of the p53 signaling pathway (33). IL-6 can affect the functions of neighboring cells by binding to the IL-6 receptor (IL-6R) signaling complex on the cell surface. The accumulation of senescent cells and the secretion of pro-inflammatory factors in senescent blood vessels can lead to changes in vasodilation, chronic inflammatory responses, and pathological extracellular matrix remodeling, causing vascular dysfunction and aging-related diseases (34).

### 3.5 Autophagy

Altered autophagy function represents a crucial molecular characteristic during the aging process of diverse organisms. Autophagy, a lysosome-mediated degradation process, serves to eliminate abnormal components within the cytoplasm, such as malfunctioning mitochondria and protein aggregates, thus maintaining cellular homeostasis. This mechanism is also intricately associated with the deceleration of the aging process. Both natural aging and pathological aging are typically accompanied by a decline in autophagic capacity (35, 36). The aging heart is regulated by multiple core metabolic sensors, including AMP-activated protein kinase (AMPK) and mammalian target of rapamycin (mTOR) (37). In aging cells, overactivation of mTOR and reduced AMPK activity can directly inhibit autophagy by inactivating the autophagy precursor ULK1 complex (38). Additionally, age-related alterations in longevity signaling pathways contribute to the transcriptional regulation of autophagy. For instance, the TF EB family of autophagy precursors is negatively regulated by mTOR-mediated phosphorylation, leading to the cytoplasmic retention and inactivation of TFs EB (39).

### 3.6 Epigenetics

Epigenetic alterations encompass DNA methylation, histone acetylation. DNA methylation with age constitutes an “epigenetic clock” that can be used as a marker of biological age. In the left ventricular tissue of patients with heart failure, there are global methylation differences in promoter CpG islands, intragenic CpG islands and gene bodies. The differential DNA methylation of DUX4 locus may be involved in the occurrence of cardiac dysfunction by reducing the survival rate of cardiomyocytes (40). AND compared with the proliferating cells, senescent cells exhibit markedly distinct methylation features: the modulation of DNA methyltransferase 1 (DNMT1) gives rise to hypomethylation in the late-replicating gene regions and hypermethylation in certain promoter-proximal regions. These modifications are hypothesized to initiate cell cycle inhibition, ultimately culminating in proliferation arrest or cells withdrawing from the cell cycle (41, 42). In addition, histone modifications not only directly modify the physical architecture of chromatin but also further govern chromatin remodeling by recruiting adaptor proteins/effector proteins bearing specific binding domains (43). Sirtuins (SIRT), a family of nicotinamide adenine dinucleotide (NAD+)-dependent histone deacetylases, play a part in retarding the senescent phenotype in diverse cell types. For instance, in cardiomyocytes, SIRT1 suppresses the transcription of genes associated with the SASP via histone deacetylation; whereas in endothelial cells, SIRT1 sustains endothelial function by regulating endothelial nitric oxide synthase (eNOS), thereby alleviating oxidative damage (44).

## 4 The molecular mechanism of transcriptional regulation in cardiovascular aging

**Figure 3:**
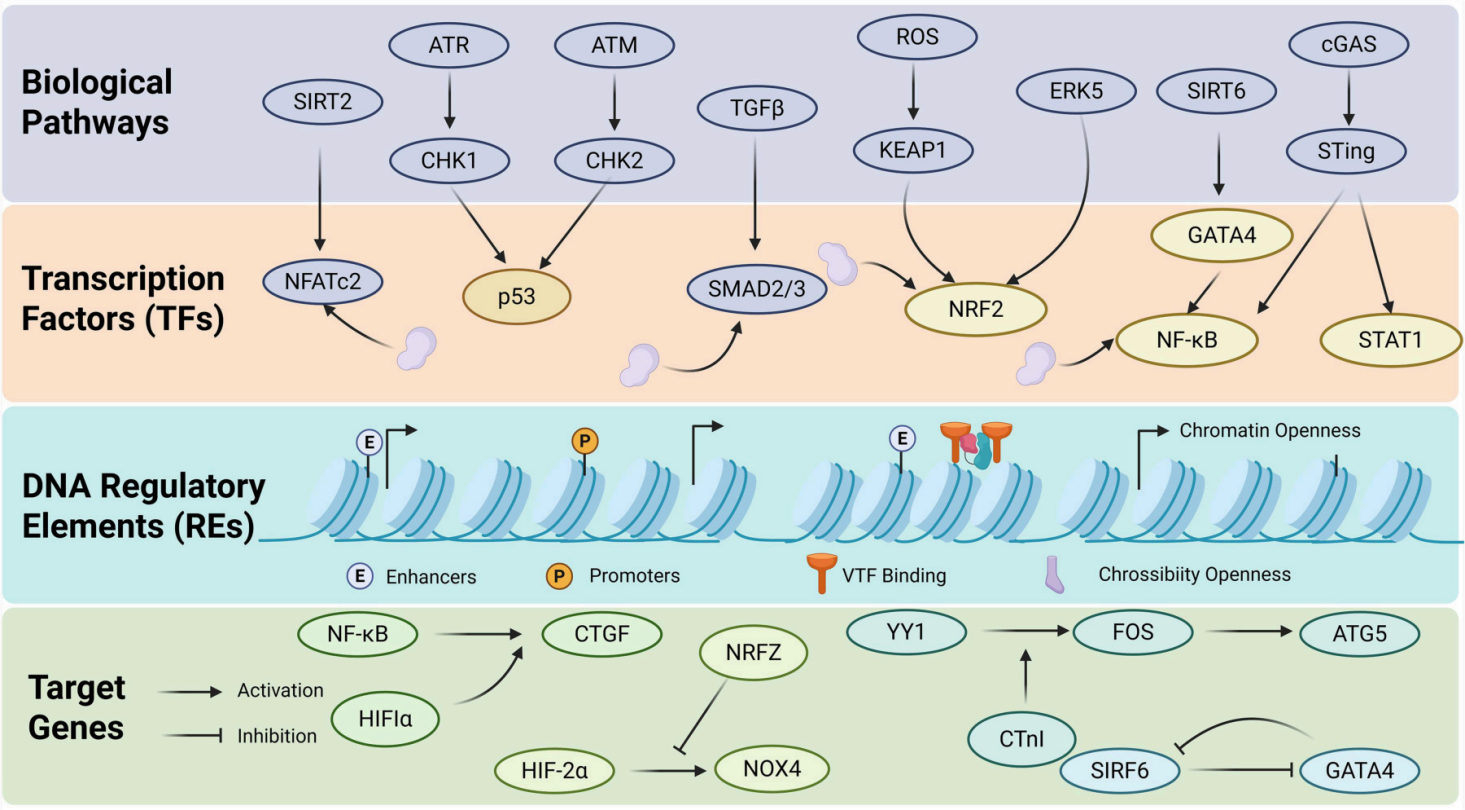
Hierarchical regulatory network of TFs in cardiovascular aging. Biological signaling pathways such as TGF*β*, ATR/CHK1, ATM/CHK2, ROS/KEAP1, ERK5, SIRT6, and cGAS/STING activate or inhibit TFs such as p53, NRF2, GATA4, SMAD2/3, NFATc2, NF-*κ*B, and STAT1 through signal cascades. These TFs also bind to the regulatory elements of DNA (such as enhancers and promoters), regulate the chromatin openness to affect the initiation of gene transcription, and ultimately regulate the expression of target genes such as CTGF, NOX4 and ATG5, thus forming a complex hierarchical regulatory network.

Cardiovascular aging does not reflect a simple, linear dysregulation of a single molecular pathway; rather, it represents a systemic, multifaceted deterioration of cellular function across diverse cardiovascular cell types. This functional decline arises from the cumulative impact of multiple endogenous and exogenous stressors and is orchestrated through the coordinated activity of numerous transcriptional regulators and epigenetic modifiers. This section aims to deeply analyze this transcriptional regulatory mechanism, in order to clarify how transcriptional regulation acts as a central hub to connect and drive different aging phenotypes, ultimately leading to cardiovascular aging (Figure 3).

### 4.1 TFs of cardiovascular aging

Although cell types in the cardiovascular system show a high degree of lineage specificity during aging, there are a series of core factors in the transcriptional regulatory network of cardiovascular aging. They act as “key hubs” to regulate cell senescence after being activated by various signals and binding to specific target genes. This section will focus on the key TFs in the process of cardiovascular aging, revealing how they act independently or in concert to jointly drive the degenerative changes in the cardiovascular system.

The forkhead box (Fox) protein family is a class of TFs with a winged helix structure in the DNA-binding domain. Fox proteins can not only regulate gene transcription by recruiting coactivators, but also directly bind to condensed chromatin to participate in its remodeling, and cooperate with other TFs to participate in transcriptional regulation, thus playing multiple roles in the process of cardiovascular aging (45). Foxp1 plays a protective role in endothelial cells, which inhibits endothelial cell senescence during the progression of atherosclerosis through transcriptional activation of IGF-1 and DUSP12, and up-regulation of Foxp1 can significantly reduce ox-LDL-induced NLRP3 activation (46–48). In addition, FOXO family members play a protective role in a variety of cell types. FOXO3 can eliminate reactive oxygen species by up-regulating catalase and SOD2, reduce oxidative stress, and delay age-related cardiac aging. Its genetic variants are significantly associated with human longevity, especially by reducing the mortality of patients with cardiometabolic diseases in late life (49, 50). FOXO1/3 also alleviated age-related cardiac fibrosis, and the FOXO3 genotype was significantly associated with a reduced risk of coronary heart disease, among which SNP rs12196996 was associated with reduced circFoxo3 expression and the risk of coronary heart disease (51–53). Of note, coronary heart disease was the only cause of death significantly associated with the lack of protective FOXO3 allele, suggesting that CVD is an important direction for clinical application of FOXO drugs (54).

As a major regulator of cellular antioxidant response, NRF2 enhances the transcription of antioxidant genes such as NQO1, SOD, and CAT by binding to antioxidant response elements in the nucleus, thereby removing reactive oxygen species and affecting the aging process (55, 56). Relevant studies have shown that age-related dysfunction of NRF2 is an important cause of ROS accumulation in the aorta of primates and rodents (57). Long non-coding RNA UCA1 promotes NRF2 expression through m6A modification, inhibits oxidative stress and ferroptosis, and prevents cardiomyocyte senescence (58). In normal VSMCs, NRF2 binds to the negative regulator Keap1 and is retained in the cytoplasm. Under the stimulation of ROS or senescence inducing factors, NRF2 dissociates from Keap1 and translocate into the nucleus, initiating antioxidant gene transcription (59). In addition, there is a more delicate regulatory mechanism in endothelial cells: mild-to-moderate glucose levels can trigger NRF2 activation through acetylation, and its Neh2 domain interacts with the PAS-A domain of HIF-2*α* in the nucleus to inhibit HIF-2*α* binding to the NOX4/p22phox promoter, thereby delaying endothelial aging (60). PCAF directly interacts with NRF2, and downregulation of PCAF can improve antioxidant activity by activating the NRF2 pathway (61). NRF2 also regulates catabolism and adenosine triphosphate production through AMPK to prolong life span, and inhibits the activation of FOXO1 and the pro-inflammatory factor NF-*κ*B, thereby delaying aging (62). These findings establish NRF2 as a central regulator of antioxidant activity in the cardiovascular system.

STAT family members STAT1 and STAT3, as key signal transduction TFs, play a central role in the process of transmitting extracellular signals from the plasma membrane to the nucleus and regulating transcription, profoundly affecting the inflammation, metabolism and aging process of the heart (63). STAT1 is particularly important in endothelial cells and cardiomyocytes, and it positively regulates the expression of STING, which exacerbates the generation of cellular SASP. STAT1 levels are significantly upregulated in mouse models of hypertensive heart failure such as HFpEF and in drug-induced cardiomyocyte senescence, directly linking signal input to inflammation (64). In contrast, ERG TFs in endothelial cells can inhibit STAT1 activity and thus attenuate SASP secretion (65). The role of STAT3 is more complex. In cardiomyocytes, an increase in nuclear location impairs mitochondrial bioenergy metabolism and accelerates the progression of heart failure (66), and its activity is regulated by both RnD3-mediated ubiquitination and degradation as well as the sulfurization modification of endogenous SO2 at Cys259 (67). In addition, alterna-day fasting has been shown to reverse the decrease in STAT3 activation and attenuate cardiac hypertrophy in aged rats (68). Given the central role of STAT1 and STAT3 in the regulation of SASP, inflammation and mitochondrial function, these two pathways have become important entry points for targeted intervention of cardiovascular aging. Related studies have confirmed that EMPA can improve heart failure in mice by inhibiting INF*γ*-STAT1-STING signaling pathway (64). STAT3 inhibitor S3I-201 can effectively block the activation of STAT3 induced by high glucose and significantly delay the senescence of cardiomyocytes (67).

Hypoxia-inducible factor 1*α* (HIF1*α*) serves as a master transcriptional regulator in hypoxic microenvironments and exerts distinct, context-dependent functions in cardiovascular aging. Unlike conventional TFs, which are primarily regulated at the transcriptional or translational levels, HIF-1*α* activity is predominantly controlled post-translationally, chiefly through oxygen-dependent regulation of its protein stability. Under normoxic conditions, HIF1*α* undergoes prolyl hydroxylation by prolyl hydroxylase domain enzymes (PHDs), enabling its recognition by the von Hippel–Lindau (VHL) E3 ubiquitin ligase complex and subsequent degradation via the ubiquitin–proteasome system. Conversely, during aging-associated hypoxia, oxidative stress, or mitochondrial dysfunction, PHD activity is attenuated, leading to HIF1*α* stabilization, nuclear translocation, and transcriptional activation of downstream target genes involved in angiogenesis, metabolic reprogramming, and cellular adaptation (69). In aged vascular tissues, HIF1*α* expression and stability are coordinately modulated by a constellation of aging-associated molecular perturbations. Beyond canonical hypoxic induction, age-related dysregulation of redox homeostasis—including diminished activity of superoxide dismutase isoforms (SOD1 and SOD2), consequent hydrogen peroxide accumulation—and elevated levels of pro-inflammatory and pro-fibrotic mediators such as platelet-derived growth factor (PDGF) and TNF*α* collectively promote HIF1*α* protein stabilization by inhibiting prolyl hydroxylase domain enzyme (PHD) activity and impairing VHL-mediated ubiquitination (69). In senescent human aortic VSMCs, HIF-1*α* differentially regulates the activities of MR-P1 and MR-1*α* transcriptional variants by binding to two cis-regulatory elements on the mineralocorticoid receptor (MR) gene promoter, leading to enhanced MR Expression under aging and oxidative stress conditions. In addition, HIF-1*α* and NF-*κ*B have a functional synergistic effect. They increase with age and together occupy the promoter region of connective tissue growth factor (CTGF) and activate its transcription process, thereby driving age-related vascular remodeling and dysfunction (70).

In addition to the above-mentioned TFs, Certain transcriptional regulatory factors also participate in the process of cardiovascular aging through specific mechanisms. Notably, the TFs involved in vascular aging and those involved in cardiac aging show significant differences in terms of tissue type and functional mechanisms. In terms of vascular aging, TFs mainly function in endothelial cells and vascular VSMCs, responding to signals such as autophagy, oxidative stress and inflammation, and regulating cellular senescence through multiple mechanisms. In terms of antioxidative stress and cell protection, MEF2A, as an important protective factor, enhances the anti-aging effect mediated by SIRT1 by directly activating the transcription of PI3 K, thereby inhibiting the senescence of endothelial cells induced by oxidative stress (71). In terms of the cell cycle and oxidative stress, CUX1 regulates the expression of CDKN2A/B by binding to the atherosclerosis-related SNP rs1537371, inducing p16-dependent senescence in endothelial cells and being closely related to oxidative stress (72). Regarding autophagy and the maintenance of cellular homeostasis, KLF4 enhances autophagy and anti-senescence ability in vascular VSMCs by up-regulating TERT expression, and antagonizes angiotensin II-induced senescence (73). Additionally, BRD4 plays a key protective role. Its deficiency disrupts the cytoskeleton and autophagy function by down-regulating the expression of tubulin and p62, leading to vascular senescence (74, 75). These factors jointly participate in the process of vascular senescence through multi-level regulatory mechanisms.

In terms of cardiac aging, transcriptional regulatory factors mainly function in cardiomyocytes, responding to signals such as genomic damage and metabolic reprogramming, and regulating the process of cardiac aging through multiple mechanisms. In terms of cell cycle and DNA damage regulation, p53, as a classical TF, has an increased binding ability to Omi/HtrA2 promoter in cardiomyocytes with aging (76), and promotes cardiac aging by directly binding to PPAR*α* promoter and inhibiting its expression (77). TBX3 protects senescent cardiomyocytes by directly regulating p21 expression, and its loss will aggravate apoptosis and mitochondrial dysfunction (78). In terms of drug-induced cardiotoxicity, YAP reverses the doxorubicin-induced senescence phenotype by activating CDK6, NRG1 promotes YAP nuclear translocation to alleviate cardiotoxicity by inhibiting LATS1/MST1 phosphorylation, and Vitpofine-blocking YAP-TEAD binding accelerates cardiac aging (79, 80).

### 4.2 Epigenetic factors

In addition, epigenetic regulatory factors, as the key link between the metabolic state of cells and the regulation of gene expression, play an increasingly important role in the aging process of the cardiovascular system. In vascular tissues, epigenetic regulation is particularly critical for maintaining the functional homeostasis of VSMCs and endothelial cells. As the third class of histone deacetylase, SIRT6 binds to the telomere region and catalyzes the deacetylation of histone H3K9 in VSMCs, ensuring the stable recruitment of telomere-associated proteins, thereby preventing replicative telomere dysfunction and cell senescenc (81, 82). Moreover, SIRT6 deletion or its H133Y mutation results in H3K9 hyperacetylation in the telomere region and induces telomere damage (83). Histone methyltransferases are also involved in the regulation of vascular senescence. EZH2, as a H3K27-specific methyltransferase, is downregulated during VSMCs senescence, resulting in a decrease in the repressive mark H3K27me3 and an increase in the activation mark H3K27ac, thereby promoting chromatin opening and transcriptional activation of aging-related genes (84).

In cardiac tissue, epigenetic regulators participate in the aging process mainly by regulating cardiomyocyte hypertrophy, inflammatory response and cell cycle exit. SIRT6 inhibits IGF-Akt and NF-*κ*B signaling pathways by deacetylating H3K9 and exerts anti-inflammatory and anti-hypertrophic effects in cardiomyocytes, thereby delaying the progression of cardiac aging and heart failure (85, 86). In addition, the two MRG15 splicing isoforms show functional differentiation during cardiac aging. MRG15S mainly regulates cell proliferation and development, and its deletion can lead to growth retardation and embryonic development disorders; However, MRG15L, as a senescence regulatory isoform, is highly expressed during terminal differentiation or senescence in tissues such as the heart and induces cell cycle arrest (87, 88). When MRG15S is replaced by MRG15L, the binding of the CDK1 promoter to acetylated histone H4 is weakened, leading to cell arrest in the G2/M phase (89). In summary, epigenetic regulators regulate vascular homeostasis and cardiac protection during cardiovascular aging through tissue-specific chromatin modification, which jointly translate changes in metabolic status into gene expression reprogramming and ultimately drive irreversible aging of the cardiovascular system.

### 4.3 Enhancer

As the core mechanism of epigenetic regulation, the activity of enhancers can be changed by epigenetic factors and TFs, thereby mediating the expression of aging-related genes and driving the process of cardiovascular aging. In endothelial cells, Smyd2 catalyzes the H3K4me1 modification, activating the distal enhancer elements adjacent to the Cdkn1a and Cdkn2a genes, thereby inducing the senescence-related phenotype (90). Notably, Foxp1, as a core TF driven by super enhancers, forms phase-separated aggregates in endothelial cells to promote SESN3 promoter binding and inhibit mTORC1 signaling, thereby delaying aging (91). Moreover, Brd4 alleviated VSMCs aging-induced hypertension by interfering with super enhancer formation that regulates angiotensin II-mediated gene expression, highlighting the complexity of enhancer regulation. Mineralocorticoid receptor reduces the trimethylation of H3K27 by reducing the expression of H3K27 methyltransferase EZH2, which is recruited to the promoter regions of stiffness genes CTGF and integrin *α*5, inducing increased acetylation of H3K27 and accelerating vascular aging (74, 75). At the level of heart tissue, TF Ets2 is also driven by ses. After activation, Ets2 can form a positive feedback regulation by binding to its own ses to promote its continuous expression, and inhibit endothelial-mesenchymal transition by regulating the expression of endothelial marker TIE1 to delay heart aging (92). p300 promotes glycolysis by increasing H3K27ac deposition at the Hk2 enhancer and participates in the metabolic remodeling of age-related cardiac aging. Moreover, drug inhibition of p300/CBP can effectively reverse the enhanced glycolysis, restore the expression of mitochondrial OXPHOS-related genes, and delay the progression of age-related cardiac dysfunction. This indicates that epigenetic intervention targeting enhancers may be a potential strategy for treating heart failure in the elderly (93). Together, these findings reveal the central role of enhancer regulation in cardiovascular aging.

### 4.4 TFs and epigenetic factors unrelated to cardiovascular aging

During the process of cardiovascular aging, although the expression or activity of a series of TFs and epigenetic regulators changes significantly with age, they do not drive aging by directly activating classic aging pathways. Instead, they indirectly shape the aging microenvironment through mechanisms such as regulating the senescence - associated secretory phenotype and mitochondrial stress response, thereby influencing the functional decline of the cardiovascular system. For instance, ATF4 activates the transcription of SMAD3 by binding to its promoter, upregulates the expression of inflammatory factors such as IL-6, and exacerbates the inflammatory response during the progression of abdominal aortic aneurysm. During mitochondrial stress, ATF5 exposes the C-terminal nuclear localization signal and translocates to the nucleus to activate the expression of antioxidant enzyme genes, thereby promoting the restoration of mitochondrial function (94). KLF2 is upregulated in response to a high - glucose environment and exerts a vascular protective effect by inhibiting the adhesion between monocytes and endothelial cells (95). NFAT3 regulates cell growth and fibrosis-related genes in response to mechanical stress and neurohormonal stimulation and participates in cardiac hypertrophy remodeling (96). In addition, SLC1A5 mRNA levels are significantly reduced in human failing myocardium, and this gene is essential for glutamine homeostasis, whose inhibition may lead to decreased glutamine uptake, which in turn may trigger metabolic disorders and aggravate heart failure progression (97). Similarly, certain CpG sites in DNA methylation clocks (such as the Horvath clock), although associated with aging, do not change due to the intrinsic aging process but are highly sensitive to glucocorticoid signaling. Synthetic glucocorticoid intervention can induce significant methylation dynamic changes at these sites, and longitudinal studies have confirmed a bidirectional association between epigenetic age acceleration (EAA) and HPA axis activity, indicating that the changes are essentially mediated by the endocrine of cortisol dynamics rather than a direct reflection of the aging process itself (98, 99). The above factors and mechanisms together constitute a complex indirect regulatory network during cardiovascular aging, emphasizing that aging-related changes may result from the accumulation of environmental and stress signals.

### 4.5 Transcriptional regulation during cardiovascular aging links aging phenotypes

Certain specific transcriptional regulators in cardiovascular aging induce cellular senescence by integrating multiple stress signals to interconnect different aspects of aging phenotypes, including genomic damage, metabolic disorders, oxidative stress, and inflammatory responses.

Telomere dysfunction and the mitochondria-inflammation axis: Telomere shortening induces chromatin uncompaction in the subtelomeric region and drives cardiomyocyte senescence by up-regulating the TF FOXC1. On the one hand, FOXC1 directly inhibits the expression of mitochondrial function-related genes, and on the other hand, it activates inflammation-related genes, thereby transforming telomere damage into mitochondrial energy metabolism disorder and cytoplasmic inflammation. Moreover, knockdown of FOXC1 can effectively inhibit the aging process and restore mitochondrial function, further confirming the central role of FOXC1 in connecting the axis of telomere dysfunction and mitochondrial inflammation (100). In addition, TGF-*β* signaling constitutes another key regulator*y* axis linking signal transduction to telomere maintenance. When activated, TGF-*β* can induce the phosphorylation and translocation of Smad2 and Smad3 to the nucleus, bind to the TERT promoter region and inhibit their transcription, resulting in decreased telomerase activity and accelerated telomere shortening (101). In contrast, Pim1 exerts a protective effect by inhibiting the phosphorylation of Smad2/3 (102). At the same time, ATF3 up-regulates the expression of Pdcd5 through the TGF*β* pathway and enhances the stability of Pdcd5 protein through the p38 MAPK pathway, thereby promoting cardiomyocyte senescence (103–105). In addition, the NFATc2-SIRT2 axis plays a key role in cardiac stress adaptation. Under basal physiological conditions, SIRT2 catalyzes the deacetylation of NFATc2, facilitating its translocation to the nucleus and subsequent proteasomal degradation. In contrast, under pathological cardiac stress conditions, SIRT2 expression is significantly reduced, leading to hyperacetylation of NFATc2, prolonged nuclear residence time, and sustained transcriptional activation of pro-aging genes (106). There is an interactive coupling between these regulators. Foxc1-mediated mitochondrial inhibition increases intracellular ROS levels, which act as second messengers to activate TGF-*β* and NFAT signaling pathways and exacerbate telomere damage and inflammation. The TGF-*β*-Smad axis inhibits TERT transcription and induces ATF3 expression, which in turn amplifies senescence-related signaling through a PDCD5-dependent mechanism. In addition, SIRT2 knockdown maintained NFATc2 activation and further enhanced the expression of pro-inflammatory genes in cardiomyocytes.

DNA damage, SASP, and autophag*y* axis: DNA damage response kinases ATM and ATR increase GATA4 protein stability and cause its accumulation in cells by inhibiting GATA4 selective autophagy. Stable GATA4 translocate into the nucleus, directly bind to the NF-*κ*B2 promoter to activate its transcription, and drive the expression and secretion of SASP factors (107). SASP factors act on adjacent cells in a paracrine manner to aggravate their DNA damage and senescence, so that the initial damage signal can be extended and continued. Under normal physiological conditions, GATA4 undergoes ubiquitination modification after binding to BMI1-RING1B, which is recognized by p62 and transported to autophagolysomes for degradation to maintain a low level of GATA4 (108, 109). However, with the decline of autophagy in the aging process, such as the impairment of the cTnI-YY1-FOS axis leading to the down-regulation of ATG5 expression (110), or the failure of the ATF3-ATG7 feedback loop leading to the blockage of autophagic flow in VSMCs (111), the ability to clear GATA4 and other pro-aging factors is weakened. So that the signal of cellular senescence can be continuously amplified. Notably, SIRT6, as a core factor in epigenetic regulation, intervenes in this connection through multiple mechanisms. Firstly, SIRT6 plays an anti-aging role by maintaining telomere stability: in VSMCs, SIRT6 binds to telomeres and promotes the deacetylation of histone H3K9 in the telomere region, and its deletion leads to H3K9 hyperacetylation, inducing telomere dysfunction and cell senescence (83). Second, SIRT6 inhibits IGF-Akt and NF-*κ*B signaling pathways by deacetylating H3K9 to prevent cardiac hypertrophy and heart failure (85). Most significantly, SIRT6 regulates the activity of GATA4 through a Tip60 - mediated acetylation switch. Initially, SIRT6 occupies the promoter region and recruits TIP60 to preserve the equilibrium of local histone acetylation. When GATA4 identifies the GATA sequence, it suppresses SIRT6 activity via interaction and stimulates TIP60 to acetylate GATA4, thereby enhancing its transcriptional activity (112). This multi - level regulatory mechanism tightly links DNA damage, autophagy, and SASP via GATA4 and SIRT6 to collectively drive the process of cardiovascular aging.

Metabolic reprogramming and epigenetic modification: During the aging process of VSMCs, the expression of mitochondrial metabolism regulator TRAP1 is significantly changed, and enhanced aerobic glycolysis leads to a large accumulation of lactic acid. On the one hand, accumulated lactate affects histone acetylation status by regulating HDAC3, and on the other hand, it directly drives histone lactatation modification. HDAC3, as a unique histone lysine deacetylase and delactinase, becomes a key regulator of H4K12 acetylation (H4K12ac) and H4K12 lactination (H4K12la) in aging VSMCs (113). TRAP1 enhanced glycolysis leading to increased lactate, which in turn promoted the accumulation of H4K12la modification by down-regulating HDAC3 activity. In addition, ChIP-seq data showed that H4K12la was significantly enriched in the SASP gene promoter region, including IL-6, IL-1*β*, MMP3, etc., which drove SASP transcription and promoted the progression of atherosclerosis (114). This mechanism expands the metabolite lactate from a traditional energy substrate to a key signal molecule for epigenetic regulation, revealing the regulator*y* axis of “metabolism-epimodify-aging”. NAD + -dependent deacetylase SIRT2 constitutes another core axis of this connection, and SIRT2 activity decreases with aging, which is closely related to the decrease in the level of its substrate NAD+ (115, 116). SIRT2 inhibits the activation of cytoplasmic mitochondrial shuttle protein p66Shc and mitochondrial reactive oxygen species (ROS) production by deacetylating its K81 site to maintain REDOX homeostasis. SIRT2 deletion results in p66Shc hyperacetylation, reactive oxygen species overload, and transcriptome reprogramming, accelerating vascular aging. Supplementation of NAD + precursor NMN can activate SIRT2 and delay vascular aging (117). Together, the two axes construct an integrated regulatory mechanism by which metabolic states link the aging transcriptional program through epigenetic modifications.

Mechanical stress and phenotypic switching: SOX9 plays a central driver role as a mechanosensitive TF during VSMCs senescence and osteogenic differentiation. SOX9 accelerates the osteogenic/cartilage phenotype transformation by regulating Extrc, and at the same time, in response to the extracellular matrix sclerosis signal, SOX9 activates the collagen-modifying enzyme LH3 to further modify matrix components and promote the increase of LH3 secretion in extracellular vesicles. With the acceleration of DNA damage and the aggravation of inflammatory response, the transmission of mechanical signals inside and outside the cell is continuously amplified (118, 119). As a key node of the pro-aging module, GATA6 expression is abnormally increased in age-related CVD such as hypertension and atherosclerosis (120, 121), which enhances its transcriptional activity by directly binding to BMP2 promoter and drives the process of osteogenic differentiation (122). The Nrf2-Id2 axis constitutes a protective connecting branch: NRF2 directly promotes ID2 transcription and inhibits the transformation of VSMCs into an osteogenic phenotype, and overexpression of ID2 can alleviate the senescence of VSMCs induced by NRF2 knockout (123). SIRT6 indirectly inhibits GATA6 transcription by promoting Nkx2-5 degradation (85, 122), and GATA6 knockdown enhances the anti-aging effect of SIRT6 and improves DNA damage repair (122). There is a complex interactive coupling between these regulators: SOX9-mediated matrix hardening further activates GATA6 expression, GATA6-driven osteogenic differentiation with increased oxidative stress inhibits the NRF2-ID2 axis, and SIRT6 regulates the balance of the upstream regulatory network by inhibiting GATA6 and maintaining telomere homeostasis. These mechanisms integrate mechanical stress, extracellular matrix remodeling, DNA damage accumulation, and inflammation into a unified pathological program that promotes the irreversible transition of VSMCs from a contractile to an osteogenic type.

### 4.6 Summary

In summary, these TFs and epigenetic factors involved in the regulation of aging collaborate with each other, jointly forming a transcriptional regulatory network that governs cardiovascular aging. At the level of vascular aging, regulation mainly occurs in endothelial cells and vascular VSMCs, focusing on oxidative stress, inflammation, autophagy and cell cycle. Among them, protective factors such as Foxp1 inhibit mTOR signaling by activating IGF-1 and phase separation to prevent atherosclerosis, and NRF2 acts as an antioxidant master regulator to remove ROS. MEF2A activates PI3 K to enhance the anti-aging effect of SIRT1, KLF4 upregulates TERT to maintain autophagy, and BRD4 stabilizes cytoskeleton and autophagy. Whereas pro-aging factors such as HIF1*α* are more stable in senescent vessels and synergization with NF-*κ*B to activate pro-fibrotic factor CTGF, ATF4 promotes inflammation through SMAD3, and CUX1 induces p16-dependent endothelial senescence.

At the level of cardiac aging, the regulation mainly acts on cardiomyocytes, focusing on DNA damage repair, mitochondrial function and metabolic reprogramming. The protective factors include FOXO3 delaying myocardial aging through anti-oxidation and anti-fibrosis, TBX3 regulating p21 to protect senescent cardiomyocytes. YAP activates CDK6 in response to drug-induced cardiotoxic activation to reverse the aging phenotype, and Ets2 inhibits cardiac endothelial-mesenchymal transition. STAT1 activates STING to aggravate inflammation; p53 enhances pro-apoptotic regulation; p38 MAPK activates Redd1 to promote fibrosis; and p300/CBP drives metabolic remodeling through epigenetic modification.

Meanwhile,the functions of specific transcription factors and epigenetic factors are highly context-dependent, and may even exhibit opposite functions under different cell types, aging stages, or stress conditions. In the case of FOXO1, although it can reduce age-related cardiac fibrosis, its overexpression in endothelial cells can lead to cell swelling and even compression of the vascular lumen (124). A similar duality is seen for other factors. A similar duality is seen for other factors. Overexpression of Nrf2 can effectively alleviate oxidative stress-induced cell senescence and play a cytoprotective role, while continuous excessive activation of Nrf2 may promote the generation of reactive oxygen species by up-regulating NADPH oxidase, accelerate cell senescence, and even enhance cancer cell survival (62). SIRT6 is widely regarded as a protective factor, its knockdown can promote atherosclerosis, but its overexpression can aggravate abnormal angiogenesis and bleeding in the plaque by activating HIF-1*α*, leading to plaque instability (125). Together, these results suggest that the function of transcription factors in cardiovascular aging is subject to dose threshold and specific pathophysiological background, and this dynamic situational dependence must be fully considered when interpreting the mechanism of action.

It is important to note that the transcriptional regulatory network of cardiovascular aging is not merely a simple list of TFs, but exhibits complex synergistic, antagonistic relationships that show remarkable specificity in different cell types. HIF1*α* and NF-*κ*B synergistically activate the expression of pro-fibrotic target gene CTGF in senescent vascular VSMCs. As a coactivator of TF YY1, cardiac troponin I (Ctni) and YY1 bind to the FOS promoter region to promote fos transcription, thereby regulating the expression of autophagy-related gene ATG5. In terms of antagonistic regulation, NRF2 directly interacts with the PAS-B domain of HIF-2*α* through its Neh2 domain in the nucleus to inhibit the binding of HIF-2*α* to the NOX4/p22phox promoter, thereby delaying endothelial cell senescence. ERG inhibited SASP factor secretion by decreasing the activity of STAT1 and NF-*κ*B. When GATA4 recognizes the promoter of its target gene, it can inhibit its deacetylase activity by interacting with SIRT6, thereby enhancing its transcription function. Together, these complex interactions constitute a complex transcriptional and epigenetic regulatory network during cardiovascular aging.

In addition, these transcriptional regulatory mechanisms closely link various aging markers, such as telomere damage, mitochondrial dysfunction, autophagy decline, metabolic disorders, and mechanical stress changes, through TFs such as FOXC1, GATA4, SOX9, and epigenetic modifications such as SIRT6, and jointly promote the irreversible functional decline of the cardiovascular system. Therefore, in-depth understanding of the transcriptional regulatory mechanisms will not only help to reveal the systemic nature of cardiovascular aging, but also provide a theoretical basis for intervention strategies targeting the key regulatory nodes.

## 5 Conclusion

The incidence of CVD increases significantly with age and has become one of the leading causes of death in the world. The cardiovascular system is responsible for delivering oxygen and nutrients throughout the body, and its aging process is accompanied by multifaceted pathophysiological changes that significantly increase disease susceptibility. A large number of studies have confirmed that cardiovascular aging is a key promoting factor for the occurrence and development of diseases such as atherosclerosis, myocardial hypertrophy and heart failure. However, due to the lack of effective intervention methods, its clinical treatment is still facing great challenges. The aging process involves complex physiological changes, among which the decline of transcriptional regulation is particularly prominent. Transcriptional regulation is the core mechanism to regulate gene expression, and its disorder will lead to the imbalance of key gene expression. This dysregulation is particularly pronounced in the cardiovascular system, where gene expression profiles of cardiac and vascular cells undergo significant remodeling, undermining basic functions such as myocardial contractility and endothelial barrier integrity, and driving progressive structural and functional deterioration of cardiovascular tissues. Thus, transcriptional dysregulation is a central mechanism driving cardiovascular aging and related pathological processes.

Although significant progress has been made in this field in recent years, there are still many key issues and limitations that need to be addressed. Firstly, current research mainly focuses on the functional study of individual TFs, while the systematic understanding of the multi-factor coordinated regulatory network is still very limited. Cardiovascular aging is the result of multiple stressors driving and the coordinated action of numerous regulatory factors, but existing research is unable to comprehensively reveal the spatiotemporal dynamic changes and interactions of these factors in specific cell types. Secondly, the issue of cell heterogeneity has not been given sufficient attention. Different subgroups within the same cell type may exhibit completely different aging trajectories, and single-cell level research is still in its infancy. Finally, the initiation and propagation mechanisms of aging signals are still unclear. How initial signals such as telomere damage and mitochondrial dysfunction are amplified through the transcriptional network and eventually evolve into a systemic aging phenotype, the molecular mechanism still needs to be further elucidated.

Moreover, TFs and epigenetic modifiers, as potential therapeutic targets, also face unique challenges in drugability and clinical translation. Firstly, constructing drugs related to TFs is the primary challenge in their clinical translation. TFs typically lack classic small - molecule binding pockets, and their functions involve complex interactions with DNA, cofactors, and chromatin - modifying enzymes, making traditional drug screening strategies ineffective (126). Secondly, there is insufficient targeting specificity. TFs play physiological roles in various tissues and cell types, and systemic intervention may lead to unpredictable off - target effects (127). For example, globally activating p53 can eliminate senescent cells but may increase the risk of tumor - suppression imbalance (128). Additionally, targeting GATA4 can inhibit SASP but may interfere with its normal function during development (129). Thirdly, Achieving cell type - specific and spatio - temporal specific epigenetic regulation remains a key challenge in clinical translation (130). Finally, the therapeutic strategies for aging intervention are not yet clear. When to intervene, which cell types to target, and which regulatory nodes to focus on to maximize therapeutic effects and minimize side effects still require systematic research.

In the face of these challenges, the development of emerging technologies has provided new approaches to solve existing problems. The application of single-cell multi-omics techniques will enable us to analyze the dynamic changes of transcriptomes, epigenomes and metabolomes of different cell types during cardiovascular aging at the single-cell resolution, revealing the role of cell heterogeneity and rare cell subpopulations in aging (131). Spatial transcriptomics technology further combines gene expression information with tissue spatial structure, providing a new perspective for understanding cell communication and the regulation of the microenvironment on aging (132). Time series analysis combined with lineage tracing technology is expected to reveal the dynamic trajectory of cell state transitions during the aging process (133). In addition, gene editing technology and its derivative tools (such as CRISPRa/CRISPRi) can conduct high-throughput screening and identification of key regulatory factors and analyze their functions and mechanisms (134).

In terms of the development of treatment strategies, multiple technical routes also show great potential. Targeted protein degradation technologies, such as protein degradation targeting chimeras (PROTAC), have opened up new directions for difficult-to-drug transcribed factors (135). By inducing the ubiquitination and degradation of TFs, they bypass the limitations of traditional small molecule inhibitors. Epigenetic drugs such as HDAC inhibitors and DNMT inhibitors have shown potential in tumor treatment (136). Their application in cardiovascular aging deserves in-depth exploration, but safety issues due to cell type specificity and long-term use need to be addressed. Metabolic intervention strategies, such as clinical research based on NAD + supplementation therapy (NMN, NR), bring hope for delaying aging-related CVD. The screening and development of SIRT2 activators are also important future directions. Lactic acid modification-related interventions, by targeting the TRAP1-HDAC3-H4K12la axis, using glycolysis inhibitors (such as 2-DG) or lactate dehydrogenase inhibitors (such as oxamate, GNE-140), have shown anti-atherosclerotic effects in animal models and are worthy of further clinical translation exploration (137). At the same time, combined intervention strategies targeting multiple core nodes, such as simultaneously activating SIRT6, inhibiting GATA4, and supplementing NAD+, may produce synergistic effects and are worthy of systematic evaluation.

In conclusion, this review has systematically summarized the key TFs and epigenetic modifiers associated with cardiovascular aging. It has also revealed that these factors may form a hierarchical regulatory network linking telomere dysfunction, mitochondrial metabolic disorders, DNA damage, loss of autophagy, metabolic reprogramming, and mechanical stress changes, all of which are hallmarks of aging. Although there are still many challenges from basic research to clinical translation, with the rapid development of single-cell multi-omics, gene editing, PROTAC technology and epigenetic drugs, targeting transcriptional regulatory networks to prevent cardiovascular aging is showing broad prospects. Future studies need to further integrate multi-omics data and functional validation to further analyze the spatiotemporal dynamic characteristics of transcriptional regulatory networks, promote cardiovascular aging research into precision medicine, and ultimately provide new therapeutic strategies for delaying cardiovascular aging and related diseases.
